# Selective elimination of senescent cells by mitochondrial targeting is regulated by ANT2

**DOI:** 10.1038/s41418-018-0118-3

**Published:** 2018-05-21

**Authors:** Sona Hubackova, Eliska Davidova, Katerina Rohlenova, Jan Stursa, Lukas Werner, Ladislav Andera, LanFeng Dong, Mikkel G. Terp, Zdenek Hodny, Henrik J. Ditzel, Jakub Rohlena, Jiri Neuzil

**Affiliations:** 1 Laboratory of Molecular Therapy, Institute of Biotechnology, Czech Academy of Sciences, Prague, 252 50 Czech Republic; 2 Laboratory of Service Technology, Institute of Biotechnology, Czech Academy of Sciences, Prague, 252 50 Czech Republic; 3 School of Medical Science, Menzies Health Institute Queensland, Griffith University, Southport, QLD 4222 Australia; 4 Department of Cancer and Inflammation Research,Institute of Molecular Medicine, University of Southern Denmark, 5000 Odense, Denmark; 5 Department of Genome Integrity, Institute of Molecular Genetics of the Academy of Sciences of the Czech Republic, v.v.i, Prague, 142 20 Czech Republic; 6 Academy of Geriatric Cancer Research (AgeCare), Department of Oncology, Odense University Hospital, 5000 Odense, Denmark; 7 Present Address: Laboratory of Angiogenesis and Vascular Metabolism, VIB-KU Leuven Center for Cancer Biology, Department of Oncology, KU Leuven, Campus Gasthuisberg O&N 4 Herestraat 49, B 912, 3000 Leuven, Belgium

**Keywords:** Cell biology, Cancer

## Abstract

Cellular senescence is a form of cell cycle arrest that limits the proliferative potential of cells, including tumour cells. However, inability of immune cells to subsequently eliminate senescent cells from the organism may lead to tissue damage, inflammation, enhanced carcinogenesis and development of age-related diseases. We found that the anticancer agent mitochondria-targeted tamoxifen (MitoTam), unlike conventional anticancer agents, kills cancer cells without inducing senescence in vitro and in vivo. Surprisingly, it also selectively eliminates both malignant and non-cancerous senescent cells. In naturally aged mice treated with MitoTam for 4 weeks, we observed a significant decrease of senescence markers in all tested organs compared to non-treated animals. Mechanistically, we found that the susceptibility of senescent cells to MitoTam is linked to a very low expression level of adenine nucleotide translocase-2 (ANT2), inherent to the senescent phenotype. Restoration of ANT2 in senescent cells resulted in resistance to MitoTam, while its downregulation in non-senescent cells promoted their MitoTam-triggered elimination. Our study documents a novel, translationally intriguing role for an anticancer agent targeting mitochondria, that may result in a new strategy for the treatment of age-related diseases and senescence-associated pathologies.

## Introduction

Cellular senescence is a complex stress-response process activated in damaged cells and resulting in permanent cell cycle arrest of affected cell [1–3]. Although, senescence-associated growth arrest counteracts cancer progression, senescent cells can also drive aging and associated pathologies through various means, including secretion of negatively acting paracrine factors collectively referred to as senescence-associated secretory phenotype (SASP) [4–7]. These factors not only contribute to the maintenance of senescence via a self-amplifying feedback loop, but can also induce senescence in healthy cells in a paracrine manner [8], for example due to increase in reactive oxygen species (ROS) [9, 10]. Accumulation of senescent cells may therefore not only accelerate ageing [11, 12], but also exacerbate progression of severe diseases such as diabetes, obesity, atherosclerosis or cataract [13–16], and promote adverse effects of chemotherapy and cancer relapse [17].

Despite irreversible cell cycle arrest, senescent cells remain metabolically active. Increased oxygen consumption, mitochondrial potential (ΔΨ_m,i_), energy production, lipid catabolism and high levels of ROS due to high-mitochondrial respiration indicate a robust metabolic shift in senescent cells [18]. The increased mitochondrial oxidative phosphorylation (OXPHOS) is crucial for the induction of oncogene-induced senescence accompanied by upregulation of PGC1α [19], which is a major regulator of mitochondrial biogenesis [20]. The mitochondrial ‘gatekeeper’ pyruvate dehydrogenase was shown to act as a crucial mediator of senescence induced by the BRAF mutant protein [19]. It was described that high production of SASP factors and senescence-related oxidative stress invoke endoplasmic reticulum stress, which promotes formation of misfolded proteins. Their repair is an energy-intensive process. Therefore, blocking the energy providing machineries of ATP synthesis leads to elimination of senescent cells in vitro [21, 22].

Adenine nucleotide translocase-2 (ANT2) belongs to the mitochondrial carrier family proteins and plays an important role mainly in translocation of ATP produced by glycolysis into mitochondria, which is critical for mitochondrial biogenesis under specific conditions [23]. Expression of ANT2 is activated during cell proliferation, and is repressed by NF1/SMAD4 complex when cells become growth arrested [24]. Overexpression of ANT2 was therefore observed in cancer cells which, unlike healthy cells, intensively employ glycolysis to support proliferation and adaptation to the intra-tumoral hypoxic conditions [25]. Downregulation of ANT2 in these cells leads to increased ROS production and apoptosis [26].

Previous studies on prematurely aged transgenic mice that induce apoptosis in p16^Ink4a^ expressing cells show correlation between senescent cell removal, delay of the ‘aged’ phenotype [11] and prolonged lifespan [12]. Furthermore, late-life clearance of senescent cells attenuates the progression of already established age-related disorders [11], as well as adaptive thermogenesis [27]. Senescent cells are often resistant to pro-apoptotic stimuli due to overexpression of the anti-apoptotic Bcl-2 protein [28]. Targeting of Bcl-2 by specific inhibitors causes apoptosis of senescent cells [29], being beneficial for the organism via ‘rejuvenation’ of aged hematopoietic stem cells [30] and restoration of tissue homoeostasis [31]. Despite these promising results, there is currently no pharmacological treatment preferentially targeting senescent cells.

Mitocans are agents with anticancer activity that induce apoptosis of malignant cells via targeting mitochondria [32]. We have developed several highly specific mitocans, which selective mitochondrial uptake is driven by high ΔΨ_m,i_ of cancer cells [33–35]. Although, these agents were intended to eliminate malignant cells, their ability to target cells with increased mitochondrial potential such as senescent cells make them intriguing candidates as possible senolytic agents. We show here that mitochondria-targeted tamoxifen (MitoTam) selectively kills senescent cells in vitro, as well as in vivo. We document that the agent suppresses OXPHOS and decreases (ΔΨ_m,i_) in senescent cells resulting in the loss of mitochondrial integrity and cell death and we show the role of ANT2 in resistance of cells to MitoTam. Our results ‘repurpose’ the anticancer agent MitoTam as a potential new clinicaly relevant drug for improving a variety of dysfunctions associated with pathological ageing.

## Results

### MitoTam effectively kills tumour cells without induction of senescence

**Fig. 1:**
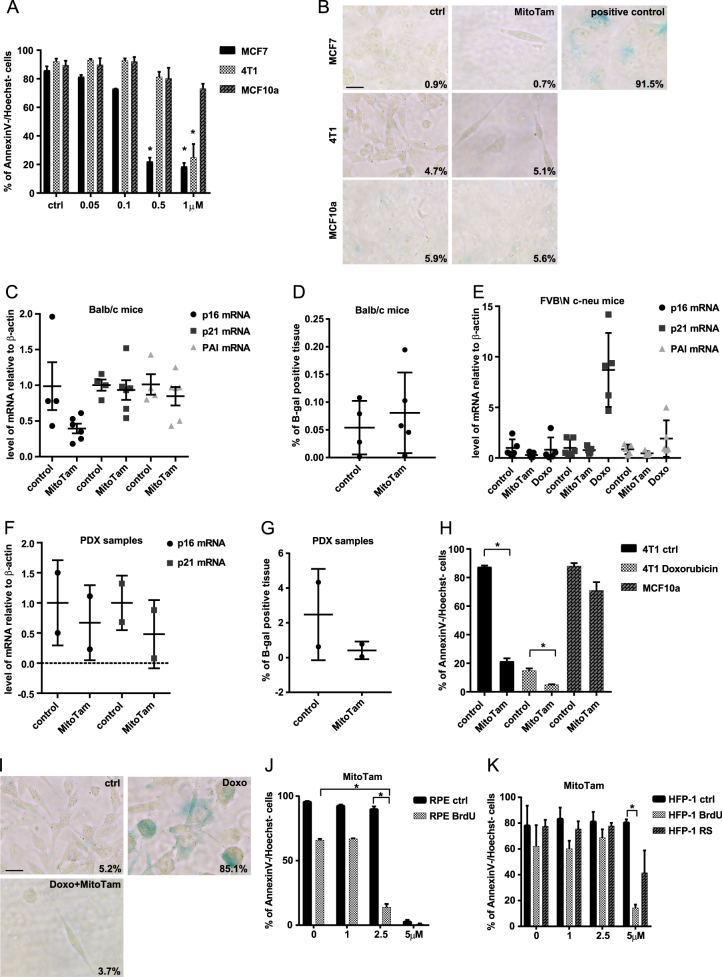
**a** MCF7, 4T1 and MCF10a cells were exposed to MitoTam for 3 days at the concentrations as indicated and cell survival was evaluated as annexin V/Hoechst negativity by flow cytometry. **b** MCF7, 4T1 and MCF10a cells were treated for 3 days with MitoTam (1 μM) and assessed for β-gal positivity. MCF7 cells treated for 8 days with 0.75 μM doxorubicine were used as a positive control of staining. Scale bar represents 10 μm. Balb-c mice were injected s.c. with 1 × 10^6^ 4T1 cells per animal and treated i.p. with MitoTam (0.25 μmol/mouse/dose) dissolved in 4% ethanol in corn oil or with the excipient twice per week for 2 weeks; the level of p16^Ink4a^, p21^waf1^ and PAI mRNA was estimated by qRT-PCR (**c**) and % of β-gal-positive tissue was detected (**d**). **e** Transgenic FVB/N c-neu mice with spontaneous tumours were treated twice per week i.p. with MitoTam (0.54 μmol/mouse/dose) dissolved in 4% ethanol in corn oil or with the excipient for 2 weeks. Mice treated with doxorubicin (1.5 mg/kg in 0.9% NaCl) for the same time were used as positive control; the level of p16^Ink4a^, p21^waf1^ and PAI mRNA was estimated by qRT-PCR. NSG mice with PDX tumours were treated with MitoTam (0.375 μmol/mouse/dose) dissolved in 4% ethanol in corn oil or with the excipient twice per week for 2 weeks. The level of p16^Ink4a^, p21^waf1^ and PAI mRNA was estimated by qRT-PCR (**f**) and % of β-gal-positive tissue was detected (**g**). **h** Control and senescent 4T1 cells (treated with 0.75 μM doxorubicin for 4 days) were exposed to 1 μM MitoTam for 3 days and cell survival was evaluated using the annexin V/Hoechst assay and flow cytometry. MCF10a cells were used as a control of toxicity. **i** 4T1 cells were treated with 0.75 μM doxorubicin for 4 days and then treated with 1 μM MitoTam for 72 h, and assessed for β-gal positivity. Scale bar represents 10 μm. RPE-1 (**j**) and HFP-1 (**k**) control and senescent (BrdU, 100 μM BrdU for 8 days; RS replicative senescence) cells were exposed to MitoTam for 48 h at the concentrations as indicated, and their survival was evaluated based on annexin V/Hoechst negativity using flow cytometry. The asterisk represents *p* < 0.05

Many established chemotherapeutics target proliferating malignant cells, which effectively blocks tumour progression. However, this is frequently accompanied by development of senescence. As MitoTam is an effective anticancer agent with promising clinical application, we first tested if it also induces cellular senescence during cancer cell treatment. When we exposed 4T1 and MCF7 breast cancer cells to MitoTam, we observed concentration-dependent induction of apoptosis, while there was no effect for non-malignant MCF10a breast epithelial cells (Fig. 1a). Cells surviving 3 days of exposure to MitoTam stained negatively for β-galactosidase, a marker of senescence (Fig. 1b). To confirm that MitoTam does not induce senescence in vivo, Balb/c mice were subcutaneously (s.c.) injected with murine breast cancer 4T1 cells and treated with the agent intraperitoneally (i.p.) twice a week. While MitoTam suppressed tumour progression [35], we did not find any significant increase in transcripts of senescence markers p16^Ink4a^, p21^waf1^ and PAI in tumours of MitoTam-treated mice (Fig. 1c) or in β-galactosidase activity (Fig. 1d). Similar results were obtained in transgenic FVB/N c-neu mice with spontaneous Her2-high breast carcinomas treated twice a week with MitoTam or doxorubicin as a control of senescence induction (Fig. 1e). To corroborate these findings, we used mice with patient-derived xenografts (PDXs) from triple-negative breast cancer, and found neither increase in transcripts of p16^Ink4a^, p21^waf1^ and PAI nor in β-galactosidase activity in tumours of MitoTam-treated mice (Fig. 1f, g). Hence, MitoTam efficiently eliminates cancer cells without inducing senescence.

During tumour development, spontaneous incidence of senescence was detected in premalignant tumours [36]. To examine if MitoTam is able to eliminate not only tumour cells but also their senescent counterparts, we induced senescence in 4T1 and MCF7 cells by treatment with doxorubicin for 4 and 8 days, respectively (see Fig. 1i and Fig. S1B for β-gal staining). To our surprise, we observed that majority of senescent cells were efficiently eliminated by MitoTam (Fig. 1h and Fig. S1A). MCF10a and retinal pigment epithelial (RPE) cells were used as a control of MitoTam toxicity for non-malignant cells. The small population of cells (about 5% of 4T1 cells and 1% of MCF7 cells) surviving MitoTam treatment did not show any increase in β-galactosidase positivity (Fig. 1i and Fig. S1B). This indicates that MitoTam is a potent anti-senolytic agent in the cancer setting.

**Fig. 2:**
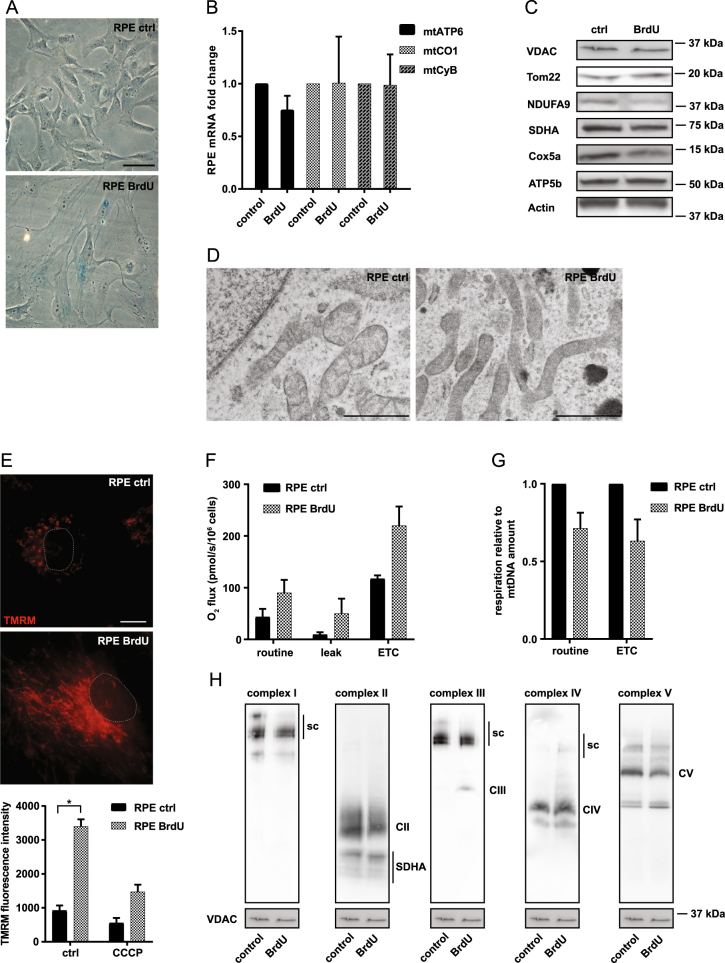
**a** RPE-1 cells were treated with 100 μM BrdU for 8 days and assessed for β-gal positivity. Scale bar represents 50 μm. **b** Expression of mtATP6, mtCO1 and mtCyB genes in RPE-1 control and senescent (BrdU) cells was detected by qRT-PCR. **c** RPE-1 control and senescent (BrdU) cells were assessed for VDAC, Tom22, NDUFA9, SDHA, Cox5a and ATP5b protein levels using immunoblotting. β-actin was used as a loading control. **d** Structure of mitochondria in RPE-1 control and senescent (BrdU) cells was imaged using transmission electron microscopy. Scale bar represents 1 μm. **e** RPE-1 control and senescent cells (BrdU) treated or not with CCCP (10 μM) were assessed for mitochondrial potential using TMRM combined with immunofluorescence microscopy (upper panel) or flow cytometry (lower panel). Scale bar represents 10 μm. **f** RPE-1 control and senescent cells (BrdU) were evaluated for routine, leak, and ETC respiration. **g** Respiration of RPE-1 control and senescent cells (BrdU) related to mtDNA amount. **h** Mitochondrial fraction from RPE-1 control and senescent (BrdU) cells was probed for respiratory complexes and supercomplexes (sc) by western blotting following NBGE using the following antibodies: CI, NDUFA9; CII, SDHA; CIII, UQCRC2; CIV, Cox5a; and CV, ATPβ; VDAC was used as loading control. The asterisk represents *p* < 0.05

Having established that MitoTam does not induce senescence in tumours and that it efficiently eliminates tumour-derived senescent cells, we investigated the effect of MitoTam on tumour-unrelated senescence. Non-malignant immortalized human RPE-1 cells and patient-derived lung fibroblasts (HFP-1) were treated for 8 days with 100 μM 5-bromo-2′-deoxyuridine (BrdU) to induce premature senescence (see Fig. 2a for β-gal staining). Human foreskin fibroblasts (BJ) were allowed to undergo 83 population doublings to enter the replicative senescence (see Fig S1D for β-gal staining). Similarly to tumour-derived senescent cells, we observed specific elimination of non-malignant senescent cells by MitoTam (Fig. 1j, k and Fig. S1C) in these non-malignant settings.

Collectively, these data document that MitoTam effectively eliminates tumour cells, including those turned senescent, without induction of additional senescence both in vitro and in vivo. Importantly, we found that MitoTam not only does not induce cellular senescence per se, but also efficiently eliminates senescent cells in general, regardless of their origin.

### Mitochondria of senescent cells are more energised

Previous experiments show the ability of MitoTam to eliminate senescent cells. Since MitoTam targets mitochondria with increased ΔΨ_m,i_, we analyzed mitochondrial changes in senescent cells. Untreated RPE-1 cells and BJ cells at 28–32 population doublings were used as controls. We found only marginal changes in the expression of mitochondrial-coded genes and in the level of mitochondrial proteins with a slight decrease in NDUFA9 (catalytic subunit of the respiratory complex I) (Fig. 2b, c; Fig. S1E and F). However, senescent cells showed altered mitochondrial morphology, indicative of a more complex mitochondrial network (Fig. 2d). We also detected increased ΔΨ_m,i_ in senescent cells (Fig. 2e; Fig. S1G), which can be a result of increased respiratory chain activity. Indeed, high-resolution respirometry revealed that senescent cells respire more than control cells, as evident from increased maximal respiratory capacity (electron transport chain; ETC) (Fig. 2f; Fig. S1H). After normalization to the amount of mtDNA, which was elevated in senescent cells (Fig. S1J), there was no difference between control and senescent cells (Fig. 2g and S1I). This suggests that higher mitochondrial content per cell is the reason for apparent increased respiration. In correlation with these results, the level of respiratory supercomplexes and the respirasome (comprising complexes CI, CIII and CIV) in mitochondria of senescent cells was not increased (Fig. 2h). These data document that senescent cells feature altered mitochondrial morphology, increased respiration on the cell per bases and higher ΔΨ_m,i_.

### Unique role of MitoTam in selective elimination of senescent cells

**Fig. 3:**
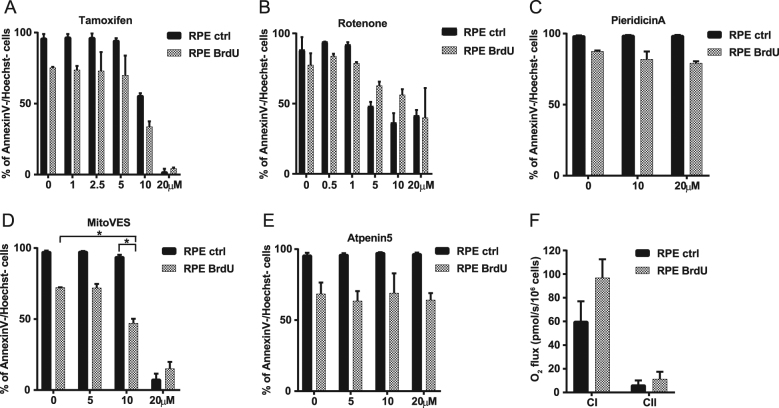
RPE-1 control and senescent (BrdU) cells were exposed to tamoxifen (**a**), rotenone (**b**), pieridicin A (**c**), MitoVES (**d**) and Atpenin5 (**e**) for 48 h at the concentrations as indicated and their survival was evaluated by annexin V/Hoechst negativity using flow cytometry. **f** RPE-1 control and senescent (BrdU) cells were evaluated for respiration via CI and CII. Data in all graphs represent means ± S.D. from three independent experiments. The asterisk represents *p* < 0.05

To examine whether effect of MitoTam on senescent cells can be replicated with other agents, we tested a range of compounds that act by inhibiting the mitochondrial function. These include agents directed against CI, the anticancer agent tamoxifen that targets CI at high concentrations [37], as well as two established inhibitors rotenone and pieridicin A. We also used inhibitors of CII, mitochondrially targeted vitamin E succinate (MitoVES) [38, 39] and atpenin 5 [40]. However, only MitoTam efficiently eliminated cells that were made senescent either by BrdU treatment or excessive replication (Fig. 3a–e; Fig. S2A-C; see Fig. 1j, k and S1C for MitoTam effect). Interestingly, we found that RPE-1 cells respire primarily via CI, which was even more pronounced in their senescent counterparts (Fig. 3f), as supported by efficient suppression of respiration by piericidin A but not by atpenin 5 (Fig. S2D, E).

To discern possible long-term toxic effects of MitoTam in normal cells, we exposed BJ cells to the agent for 2 days, at which stage we observed no significant increase of DNA damage as detected by 53BP1 and γH2AX markers (Fig. S2F). We observed about 50% inhibition of proliferation of the cells exposed to MitoTam (2.5 μM; Fig. S2G), however, without obvious complete arrest of cell proliferation. The low level of toxicity of MitoTam to non-senescent BJ cells resembles its selectivity for cancer cells [35]. Collectively, these results document unique ability of MitoTam to selectively eliminate senescent cells without noticeable toxic effects on normal cells.

### MitoTam suppresses senescence in vivo

**Fig. 4:**
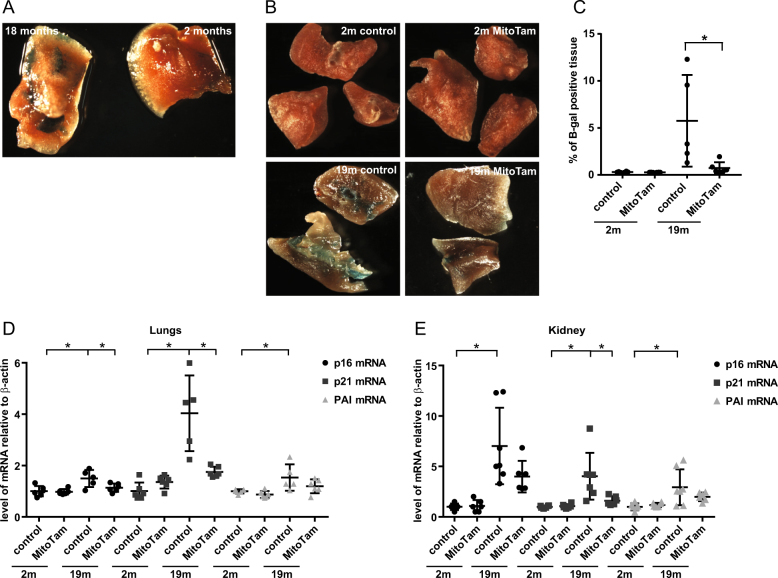
**a** Lungs from 2- and 18-month-old FVB/N mice were excised, cut into small pieces (2–3 mm^3^) and assessed for β-gal positivity (blue colour). **b** 2- and 18-month-old FVB/N mice were treated once a week i.p. with MitoTam (2 μg of MitoTam/1 g of mouse) dissolved in 4% EtOH in corn oil or solvent control with the excipient for 4 weeks, lungs were excised, cut into small pieces (2–3 mm^3^) and assessed for β-gal activity (blue colour). A length of 80 μm thin sections were then statistically analysed for β-gal positivity (**c**). Expression of p16^Ink4a^, p21^waf1^ and PAI genes in lungs (**d**) and kidney (**e**) were estimated by qRT-PCR. The asterisk represents *p* < 0.05

As MitoTam efficiently eliminates senescent cells in vitro, we next tested the senolytic activity of MitoTam in vivo in the non-malignant setting, using naturally aged FVB/N mice (18-month-old) and control 2-month-old mice. First, we assessed presence of senescent cells using β-gal staining. Of all tested organs (brain, liver, lungs, kidney, white adipose tissue and stomach), the most pronounced difference in β-gal staining between young and aged mice was detected in lungs (Fig. 4a). We next treated young and aged mice with MitoTam (2 μg/g mouse) administered once a week for a period of 4 weeks. We observed that β-gal staining of lungs from MitoTam-treated mice decreased almost to the level of control animals (Fig. 4b, c). This was accompanied by a significant decrease in the expression of senescence markers p16^Ink4a^, p21^waf1^ and PAI almost to their levels in control mice (Fig. 4d, e). Single dose of MitoTam (one dose per one week) had no significant effect on elimination of senescent cells (Fig. S3A-D). These findings reveal that MitoTam effectively eliminates senescent cells also in vivo.

### Increased level of ROS formation is not responsible for elimination of senescent cells by MitoTam

Our previous data revealed that ROS are involved in the killing of breast cancer cells by MitoTam [35]. We therefore tested whether this also applies to the elimination of senescent cells by the agent. Although, we observed increased ROS production in response to MitoTam in senescent cells (Fig. S4E, F), these cells were not protected from MitoTam-triggered killing by ROS scavenger *N*-acetylcysteine (Fig. S4A, B). To corroborate these data, we treated senescent RPE-1 and BJ cells with phenylethyl isothiocyanate (PEITC), an agent that depletes glutathione and increases ROS by a mechanism unrelated to that of MitoTam. While PEITC caused increased ROS in senescent cells (Fig. S4D), it did not affect their viability (Fig. S4C). Finally, we found similar effect of MitoVES on ROS generation in senescent cells (Fig. S4E, F), although MitoVES at this concentration (2.5 μM) had no effect on their viability (Fig. 3d and Fig. S2C). Taken together, while MitoTam causes generation of ROS in senescent cells, its ability to eliminate senescent cells is accomplished by an ROS-independent mechanism.

### Reduced ANT2 plays a role in vulnerability of senescent cells to MitoTam

**Fig. 5:**
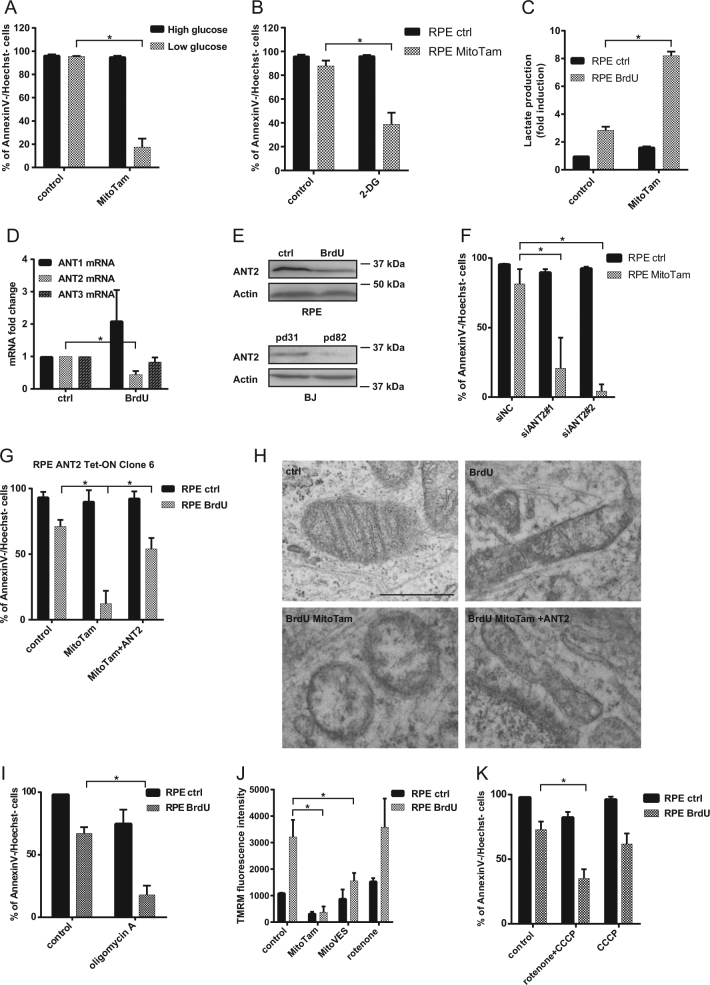
RPE-1 control cells were exposed to MitoTam (2.5 μM) for 48 h in high (4.5 g/L) and low (1 g/L) glucose medium (**a**) or in the presence of 2-deoxyglucose (2-DG; 25 mM) (**b**), and cell survival was evaluated by annexin V/Hoechst negativity using flow cytometry. **c** RPE-1 control and senescent cells (BrdU) were exposed to MitoTam (2.5 μM) for 48 h and lactate level was assessed. **d** Expression of ANT1, ANT2 and ANT3 transcripts in RPE-1 control and senescent (BrdU) cells were detected by qRT-PCR. **e** Control and senescent (BrdU) RPE-1 cells, as well as BJ control (pd 31) and senescent (pd 82) cells were assessed for the level of the ANT2 protein by western blotting. β-actin was used as loading control. **f** RPE-1 control cells were exposed to MitoTam (2.5 μM) for 48 h after downregulation of ANT2 using specific siRNAs, and their survival was evaluated by annexin V/Hoechst negativity using flow cytometry. **g** RPE-1 control and senescent (BrdU) cells, as well as their ANT2-overexpressing counterparts, were exposed to MitoTam (2.5 μM) for 48 h and viability was evaluated as annexin V/Hoechst negativity by flow cytometry. **h** Structure of mitochondria in RPE-1 control and senescent (BrdU) cells as well as their ANT2-overexpressing counterparts exposed to MitoTam (2.5 μM) for 24 h were assessed for mitochondrial morphology using transmission electron microscope. Scale bar represents 1 μm. **i** RPE-1 control and senescent (BrdU) cells were exposed to oligomycin A (5 μM) for 48 h and their survival was evaluated by annexin V/Hoechst negativity using flow cytometry. **j** Control and senescent (BrdU) RPE-1 cells were exposed to MitoTam (2.5 μM), MitoVES (2.5 μM) or rotenone (0.5 μM) for 24 h, and the cells were assessed for ΔΨ_m,i_ using TMRM and flow cytometry. **k** RPE-1 control and senescent (BrdU) cells were exposed to rotenone (0.5 μM) in combination with CCCP (10 μM) for 48 h and their survival was evaluated by annexin V/Hoechst negativity using flow cytometry. Data in all graphs represent means ± S.D. from three independent experiments. The asterisk represents *p* < 0.05

MitoTam was designed to efficiently target CI-dependent respiration. Even though RPE-1 and BJ cells respire primarily via this complex (Fig. 3f), other CI inhibitors were unable to eliminate senescent cells. This indicates that CI inhibition alone cannot explain the senolytic effect of MitoTam and that an additional mechanism is likely involved. Resistance of control cells to MitoTam points to the availability of an alternative source of ATP, since MitoTam almost completely suppressed routine respiration in RPE-1 and BJ control cells (Fig. S5A). To test if glycolysis can compensated for mitochondrial ATP production in these cells, we exposed control RPE-1 cells to MitoTam at different concentrations of glucose and found increased toxicity of this agent for the control cells cultivated at low glucose medium (Fig. 5a). Similarly, inhibition of glycolysis using 2-deoxy-d-glucose increased sensitivity of control cells cultivated at high glucose to MitoTam (Fig. 5b). However, a switch to glycolysis in response to MitoTam (2.5 μM) treatment was mild in control and robust in senescent cells, indicating that senescent cells efficiently utilize glycolysis as an alternative source of ATP (Fig. 5c), discounting inflexibility of ATP production as the reason for the susceptibility to MitoTam.

ANT proteins mediate the exchange of ADP and ATP across the inner mitochondrial membrane, playing an essential role in cellular energy metabolism. Unlike ANT1 and ANT3, which import ADP and export ATP when OXPHOS is functioning normally, ANT2 works in the opposite way and imports ATP generated via glycolysis into the mitochondrial matrix when OXPHOS is impaired. ATP is then cleaved by the ATPase activity of complex V to ADP and P_i_, contributing to the maintenance of ΔΨ_m,i_, which is necessary for cell survival and proliferation. In this context, we found that senescent cells increase level of ANT1 (Fig. 5d, Fig. S5B, C), consistent with higher respiration, and decrease level of ANT2 in vitro (Fig. 5d, e, Fig. S5B) and in vivo (Fig. S5D). Moreover, knock-down of ANT2 in control (non-senescent) cells (Fig. S5E, G) sensitized them to MitoTam (Fig. 5f, Fig. S5F).

To prove the importance of ANT2 in modulation of their response to MitoTam, we transduced senescent cells with recombinant lentiviral plasmid for inducible expressing of ANT2, and selected three clones with the highest expression level (Fig. S6A). We found that senescent cells with increased ANT2 were considerably more resistant to MitoTam treatment compared to their counterparts with low ANT2 (Fig. 5g, Fig. S6B, C) (see Fig. S6D for ANT2 Tet-ON clone 6 expression efficacy). Using electron microscopy, we observed a loss of cristae and altered morphology in mitochondria of senescent cells exposed to MitoTam, which was restored as a result of ANT2 overexpression (Fig. 5h). ANT2 and mitochondrial ATP synthase are functionally interlinked. Interestingly, blocking of ATP synthase activity by oligomycin A resulted in decreased viability of senescent cells (Fig. 5i), as well as ANT2 knocked-downed control cells (Fig. S6E), recapitulating the MitoTam results.

In comparison with other mitochondrial inhibitors, we detected rapid decrease of ΔΨ_m,i_ in cells treated with MitoTam (Fig. 5j). After uncoupling of respiratory chain by carbonyl cyanide 3-chlorophenylhydrazone (CCCP) leading to reduction of ΔΨ_m,i_, we observed decreased viability of senescent cells treated with rotenone (Fig. 5k), which indicate a coupling effect of lowering ΔΨ_m,I_ and inhibition of ATPase after MitoTam treatment, since CCCP or rotenone treatment alone is not able to significantly eliminate senescent cells (Figs. 3b and 5k). Similar results were observed in ANT2 knocked-down control cells treated with CCCP (Fig. S6F). More effective decrease of viability after CCCP treatment in these cells could result from more efficient downregulation of ANT2 after siRNA in comparison with its level in senescent cells.

Together, these results document an important role for the ANT2 protein in the regulation of selective susceptibility of senescent cells to MitoTam, as well as a cumulative effect of ΔΨ_m,i_ decrease and inhibition of respiration on senescent cells after MitoTam treatment.

## Discussion

Accumulation of senescent cells leads to ageing, and exacerbates the progression of severe ageing-associated diseases and pathologies [41]. To eliminate senescent cells pharmacologically, we need to understand how their viability is maintained on the molecular level. Besides the resistance to apoptosis due to the overexpression of anti-apoptotic proteins or downregulation of caspase-3 [42, 43], these cells undergo considerable changes in their metabolism, since senescence is an energetically demanding process requiring a steady supply of ATP [18, 44]. The key finding presented herein therefore is that simultaneous interference with mitochondrial integrity and ATP haemostasis in senescent cells leads to their effective removal.

Consistent with other reports, we find that senescent cells rebuild their mitochondria into a complex network resulting in increased respiration and ΔΨ_m,i_. Importantly, we now also document that mitochondria destabilization by specific targeting that blocks cellular respiration and maintenance of ΔΨ_m,i_ selectively eliminates not only tumour-derived senescent cells, but also senescent cells in general. Senescent RPE-1 and BJ cells rely largely on respiration via CI, and of the tested agents only MitoTam, an inhibitor of respiration via CI developed originally in our laboratory as a selective anticancer agent, efficiently eliminated senescent cells in vitro and in vivo without toxic effects towards normal cells. The latter follows from experiments with naturally aged mice administered with MitoTam for 4 weeks, which resulted in significant suppression of senescence markers in all tested organs. Compared to the BH3 mimetic ABT737, MitoTam was found some four times more efficient in elimination of senescent cells (Fig. S6G). Thus, apart from the previously published studies showing senolytic activity of ABT-373 [29] and ABT-263 [30] in models of radiation-induced senescence or transgenic model of senescence, respectively, this is the first demonstration of a mitochondrial targeted pharmacologically relevant agent that has senolytic activity in vivo, pointing to a new strategy for elimination of senescent cells.

The mechanism of MitoTam-induced cell death in senescent cells is undoubtedly complex. It involves ANT2, an ANT-family member that imports ATP into mitochondria in situations of mitochondrial ATP imbalance, such as during rapid proliferation. We found that ANT2 expression is selectively reduced in senescent cells, likely a result of increased TGFβ, which is a part of the SASP [45], and which was previously found to negatively regulate ANT2 [26, 46]. The role of ANT2 reduction in MitoTam-induced cell death in senescent cells is clearly indicated by ANT2 reconstitution experiments, where ANT2 recovery resulted in resistance to MitoTam treatment. The ANT2 involvement is probably not a simple matter of ATP import into mitochondria when CI function is blocked by MitoTam. Two other unrelated CI inhibitors, rotenone and pieredicin A, did not induced cell death in senescent cells, even though at concentrations used they should completely inhibit CI and interfere with CI-driven electron transfer and proton pumping. On the other hand, ATP synthase inhibitor oligomycin A did induce cell death in senescent cells, suggesting that ATP synthase activity (either forward or reverse) is crucial to maintain mitochondrial function in the absence of ANT2.

Treatment with MitoTam, unlike with other ETC inhibitors, effectively reduced mitochondrial membrane potential in senescent cells, and severely affected mitochondrial morphology as judged by EM, which was corrected by ANT2 reconstitution. Hence, it appears that ATP synthase activity may be required to maintain mitochondrial integrity in senescent cells featuring low ANT2, particularly in a situation when the integrity of the mitochondrial network is challenged by MitoTam. In support of this conclusion, combining rotenone with the mitochondrial uncoupler CCCP also induced cell death in senescent cells, and this treatment is also expected to affect mitochondrial integrity and morphology. Furthermore, reduction of ANT2 in non-senescent cells by siRNA sensitized these cells to both MitoTam and oligomycin A, as well as CCCP. This underscores the crucial importance of the interplay between ANT2 and ATP synthase in the maintenance of mitochondrial integrity in a situation when ΔΨ_m,i_ is compromised, and reveals a therapeutic vulnerability specific to senescent cells.

A prominent consequence of ETC blockade is the induction of ROS. While ROS are pivotal for cell death induction upon application of MitoTam and other ETC inhibitors in many situations [35, 39, 47], this does not seem to be the case in senescent cells. An increase of ROS level, for example, after alteration of cellular metabolism, interference with the respiratory chain or suppression of mitochondrial potential, has been demonstrated as possibly critical for induction and maintenance of cellular senescence. Consequently, exacerbation of these perturbations could contribute to the induction of cell death [48, 49]. To our surprise, even though MitoTam increased ROS, their role in elimination of senescent cells is unlikely, since pretreatment of cells with the ROS scavenger *N*-acetylcystein had no effect on decreased viability of MitoTam-treated cells. Further, we observed increased ROS production also upon treatment with other mitochondrial inhibitors that have no effect on cell viability. It is possible, therefore, that senescent cells are better able to withstand the ROS insult, and this aspect of MitoTam activity is therefore less important.

Collectively, our results show that MitoTam very effectively kills both primary senescent cells accumulated in aging organs, as well as prematurely senescent cells, which may occur in younger organisms in response to genetic mutations, environmental influences or various pathological states. Based on the above findings and the fact that MitoTam is an experimental anticancer agent entering phase I clinical trial with an excellent toxicity profile from preclinical testing, we postulate that MitoTam, our proprietary drug, may be clinically used to eliminate senescent cells in the context of pathological senescence, as well as senescence-associated diseases.

## Material and methods

### Chemicals and antibodies

All chemicals were purchased from Sigma (St. Louis, MO, USA). For immunoblotting, the following antibodies were used: anti-ANT2, anti-cleaved caspase-3 (Cell Signaling Technology, Danvers, MA, USA), anti-Tom20, anti-cytochrome C, anti-53BP1 (Santa Cruz Biotechnology, Dallas, TX, USA), anti-ATP5B (Sigma, St. Louis, MO, USA), anti-γH2AX (Millipore, Billerica, MA, USA), anti-NDUFA9, anti-SDHA, anti-Cox5a, anti-UQCRC2, anti-ANT1 and anti-VDAC (Abcam, Cambridge, UK). All antibodies were diluted 1:1000 in 2.5% non-fat milk. Horseradish peroxidase (HRP) conjugated β-actin (ThermoFisher, Waltham, MA, USA) was used as a loading control. IgG-HRP anti-rabbit (170-6515) and anti-mouse (170-6516) secondary antibodies produced in goat were purchased from BioRad Laboratories (Hercules, CA, USA). Secondary antibodies were diluted 1:10,000 in 2.5% non-fat milk.

### Cell culture

Human breast carcinoma cell line MCF7, telomerase-immortalized human RPE-1, foreskin fibroblast cell line (BJ) and lung-derived fibroblast cell line (HFP-1) were cultivated in DMEM containing 4.5 g/l glucose (Biochrom, Berlin, Germany). The media was supplemented with 10% foetal bovine serum (Gibco, Carlsbad, CA, USA) or 10% TET-free serum (for RPE-1 Tet-ON ANT2 transfected cells), and 100 U/ml penicillin and 100 μg/ml streptomycin sulfate (Sigma, St. Louis, MO, USA). Cells were kept at 37 °C under 5% CO_2_.

### Animal studies

Transgenic FVB/N c-neu mice that developed tumours spontaneously (450 mm^3^ on average) were treated with MitoTam (0.54 μmol/mouse/dose) or solvent control (4% ethanol in corn oil, 100 μl per dose) given i.p. twice a week for 2 weeks. Balb/c mice were s.c. injected with 1 × 10^6^ 4T1 cells. After 1 week (when tumours reached on average 100 mm^3^), MitoTam (0.25 μmol/mouse/dose) or solvent control (4% ethanol in corn oil, 100 μl per dose) was administered as above for 2 weeks. The mice were killed, organs were collected and analysed.

NSG mice were implanted with patient tumour tissue, grown as first generation xenograft in mammary fat pad. Mice were anaesthetised, the mammary fat pad was surgically exposed and injected with 50 µl of Matrigel extracellular matrix (Corning, Wiesbaden, Germany). When the Matrigel solidified, tumour pieces (~1 mm^3^) were implanted into a pocket excised in the mammary fat pad and secured with an internal stitch. The incision was closed by suture and mice were left on a heated pad until awaken. When tumours reached the volume of ~50 mm^3^, mice (*n* = 2 per group) were treated with either MitoTam (0.375 µmol/mouse/dose) or the excipient (4% ethanol in corn oil, 100 µl per dose) given i.p. twice a week. At the end of the experiment, tumours were removed and analysed. All experiments were approved by the Czech academy of Sciences Ethics Committee and performed according to the Czech Council guidelines for the Care and Use of Animals in Research and Teaching.

### Assessment of respiration

Routine respiration in cells was assessed using the high-resolution Oxygraph-2k respirometer (Oroboros Instruments, Innsbruck, Austria) according to the standard procedure [34, 35]. For CI- and CII-dependent respiration, digitonin-permeabilized cells suspended in mitochondrial respiration medium were used. The total oxygen concentration and consumption were monitored in the presence of specific inhibitors of CI (rotenone) or CII (malonate).

### Evaluation of mitochondrial membrane potential and ROS

To assess ΔΨ_m,I_ and ROS production, cells were treated with tetramethylrhodamine methyl ester (TMRM; 50 nM) and 2′7′-dichlorofluorescein (DCF; 10 μM), respectively, for 30 min prior to analysis by flow cytometry (BD LSRFortessa, San Jose, CA, USA). Cells without added TMRM or DCF probes were used as a control of nonspecific signal. Hoechst 33258 (5 μg/ml) was added to cells prior to the measurements to exclude dead cells from the analysis.

### Detection of cell viability

The medium containing dead cells was collected into clear tube, adherent cells were trypsinized, resuspended in the collected medium and centrifuged at 1000×*g* for 3 min. The pellet was resuspeneded in 200 μl of annexin V buffer containing 0.3 µl of annexinV-Dyomics 647(Apronex, Vestec, Czech Republic), and incubated for 20 min at 4 °C. Hoechst 33258 (5 μg/ml, Invitrogen, Carlsbad, CA, USA) was added before analysis. The cells were analyzed for viability using the LSRFortessa flow cytometer (San Jose, CA, USA). Changes in cellular viability were expressed as the percent of the annexinV negative/Hoechst negative fraction.

### SDS-PAGE, NBGE and immunoblotting

Cells were washed twice with PBS, collected into Laemmli SDS sample lysis buffer (2% SDS, 50 mM Tris-Cl, 10% glycerol in double distilled H_2_O) and sonicated (2 × 15 s at 1 μ amplitude with 15 s cooling interval) using Soniprep 150 (MSE, London, UK). Protein concentration was estimated using the BCA method (Pierce Biotechnology, IL, Rockford, USA). Cell lysates were supplemented with 100 mM DTT and 0.01% bromophenol blue before separation by SDS-PAGE. The same amount of protein (50–70 μg) was loaded into each well. Proteins were transferred onto a nitrocellulose membrane using wet transfer and detected by specific antibodies combined with horseradish peroxidase-conjugated secondary antibodies (goat anti-rabbit, goat anti-mouse). Peroxidase activity was detected by SuperSignal West Femto Extended Duration Substrate (Thermo Fisher Scientific, Waltham, MA, USA). β-actin was used as a loading standard. Native blue gel electrophoresis was performed as described [50].

### Detection of senescence-associated beta-galactosidase activity

SA-β-gal activity was detected as previously described [51] with slight modifications. Cells were washed once with PBS, fixed with 0.5% glutaraldehyde (in PBS; pH 7.2), and washed in PBS (pH 6.0) supplemented with 1 mM MgCl_2_. Cells were stained with the X-gal solution (1 mg/ml X-gal, 0.12 mM K_3_Fe[CN]_6_, 0.12 mM K_4_Fe[CN]_6_, 1 mM MgCl_2_ in PBS at pH 6.0) at 37 °C for 3–5 h. For tissue staining, tissue was cut into small pieces (2–3 mm^3^) and fixed in 1% formaldehyde/0.2% glutaraldehyde at 4 °C for 1 h. Tissue was stained with the X-gal solution as described above. For statistical evaluation, tissue was cut into 80 μm sections. β-galactosidase signal was detected using light microscope (Leica, Mannheim, Germany) and evaluated using the Photoshop and ImageJ programme as an average from five sections/sample.

### Indirect immunofluorescence

Cells grown on glass coverslips were fixed with 4% formaldehyde and permeabilized with 0.1% Triton X-100 in two consecutive steps, each at room temperature for 15 min. After washing with PBS, cells were incubated in 10% FBS (diluted in PBS) for 30 min to block unspecific signals. After this step, cells were incubated with diluted primary antibodies at room temperature for 1 h and then extensively washed with PBS/0.1% Tween 20. The incubation with secondary antibodies was performed at room temperature for 1 h. To counterstain nuclei, coverslips were mounted in Mowiol containing 4′,6-diamidino-2-phenylindole (Sigma) and viewed by a fluorescence microscope (Leica DMRXA).

### siRNA-mediated gene knock-down

Cells were transfected with siRNAs using Lipofectamine RNAiMAX (Invitrogen, Carlsbad, CA, USA) following the manufacturer´s instructions. siRNA against ANT2 (sense sequence: ANT2#1: 5′-GCU UUA ACG UGU CUG UGC Att-3′; ANT2#2: 5′-GCU UUA ACG UGU CUG UGC Att-3′) was purchased from Applied Biosystems (Foster City, CA, USA). Non-targeting siRNA (Silencer® Select Negative Control No. 1, #4390843) were used as a negative control (siNC).

### Quantitative real time PCR (qRT-PCR)

Total RNA was isolated using RNAzol (400 μl for a 4 cm^2^ dish; Molecular Research Center, Cincinnati, OH, USA). First strand cDNA was synthesized from 1 μg of total RNA with random hexamer primers using Revert Aid First strand cDNA Synthesis Kit (Thermo Scientific, Waltham, MA USA). qRT-PCR was performed using the Eco Real-Time PCR System (Illumina, San Diego, CA, USA) with 5× HOT FIREPol Evagreen qPCR Supermix GreenE dye (Solis Biodyne, Tartu, Estonia). The relative quantity of cDNA was estimated by the ΔΔCT method, data were normalized to β-actin. The following primers were purchased from Sigma: ANT1: 5′-GCT GCC TAC TTC GGA GTC TAT G-3′, 5′-TGC GAC TGC CGT CAC ACT CTG-3′; ANT2: 5′-GCC GCC TAC TTC GGT ATC TAT G-3′, 5′-CAG CAG TGA CAG TCT GTG CGA T-3′; ANT3: 5′-GGT GAA GAT CAC CAA GTC CGA C-3′, 5′-ACC ACG ATG TGC GTG TTC TTG G-3′; mtATP6: 5′-CGC CAC CCT AGC AAT ATC AA-3′, 5′-TTA AGG CGA CAG CGA TTT CT-3′; mtCO1: 5′-TCT CAG GCT ACA CCC TAG ACC A-3′, 5′-ATC GGG GTA GTC CGA GTA ACG T-3′; mtCyB 5′-GGG GCC ACA GTA ATT ACA AA-3′, 5′-GGG GGT TGT TTG ATC CCG TTT-3′; human p16: 5′-CTC GTG CTG ATG CTA CTG AGG A-3′, 5′-GGT CGG CGC AGT TGG GCT CC-3′; human p21: 5′-TCA CTG TCT TGT ACC CTT GTG C-3′, 5′-GGC GTT TGG AGT GGT AGA AA-3′; mouse p16: 5′-TGT TGA GGC TAG AGA GGA TCT TG-3′, 5′-CGA ATC TGC ACC GTA GTT GAG C-3′; mouse p21: 5′-TCG CTG TCT TGC ACT CTG GTG T-3′, 5′-CGA ATC TGC ACC GTA GTT GAG C-3′; mouse PAI: 5′-CCT CTT CCA CAA GTC TGA TGG C-3′, 5′-GCA GTT CCA CAA CGT CAT ACT CG-3′; human β-actin: 5′-CCA ACC GCG AGA AGA TGA-3′, 5′-CCA GAG GCG TAC AGG GAT AG-3′; human mtDNA: 5′-CTG TTC CCC AAC CTT TTC C -3′, 5′-CCA TGA TTG TGA GGG GTA GG-3′; human nDNA: 5′- GCT GGG TAG CTC TAA ACA ATG TAT TCA-3′, 5′-CCA TGT ACT AAC AAA TGT CTA AAA TGG T-3′. Data are expressed as means ± S.D. of a minimum of three independent experiments performed in triplicates. The *p-*values were calculated using two-tailed Student’s *t*-test; differences with *p* < 0.05 were considered statistically significant.

### Lactate assays

For lactate assessment, 1 μl of medium was used for the analysis using a colorimetric kit (Trinity Biotech, Bray, Ireland) following 10 min incubation. Results were normalized to the number of evaluated cells.

### DNA constructs and cell transfections

Human *ANT2* cDNA was synthetized in vitro (Genscript) and subcloned into the lentiviral, doxycycline-inducible vector pLVX-TetONE. The recombinant lentiviruses were obtained from HEK293T/17 cell co-transfected with pLVX-TetONE-hANT2, psPAX2 and pMD2.G (both obtained from Addgene) by lipofectamine LF3000 (Invitrogen). Viral particles were concentrated using PEG-It reagent (SBI), dissolved in PBS and stored at −80 °C. RPE cells with inducible expression of ANT2 were prepared by lentiviral transduction and clonal selection. ANT2 expression was activated by doxycycline (1 μg/ml) 24 h before experiment.

## Electronic supplementary material

Supplementary Figure 1

Supplementary Figure 2

Supplementary Figure 3

Supplementary Figure 4

Supplementary Figure 5

Supplementary Figure 6

Supplementary legend
