# Supplementary figures and images for: Selective elimination of senescent cells by mitochondrial targeting is regulated by ANT2
Source: Cell Death Differ. 2018 May 21;26(2):276–90. doi: 10.1038/s41418-018-0118-3 (PMC6329828; doi:10.1038/s41418-018-0118-3)

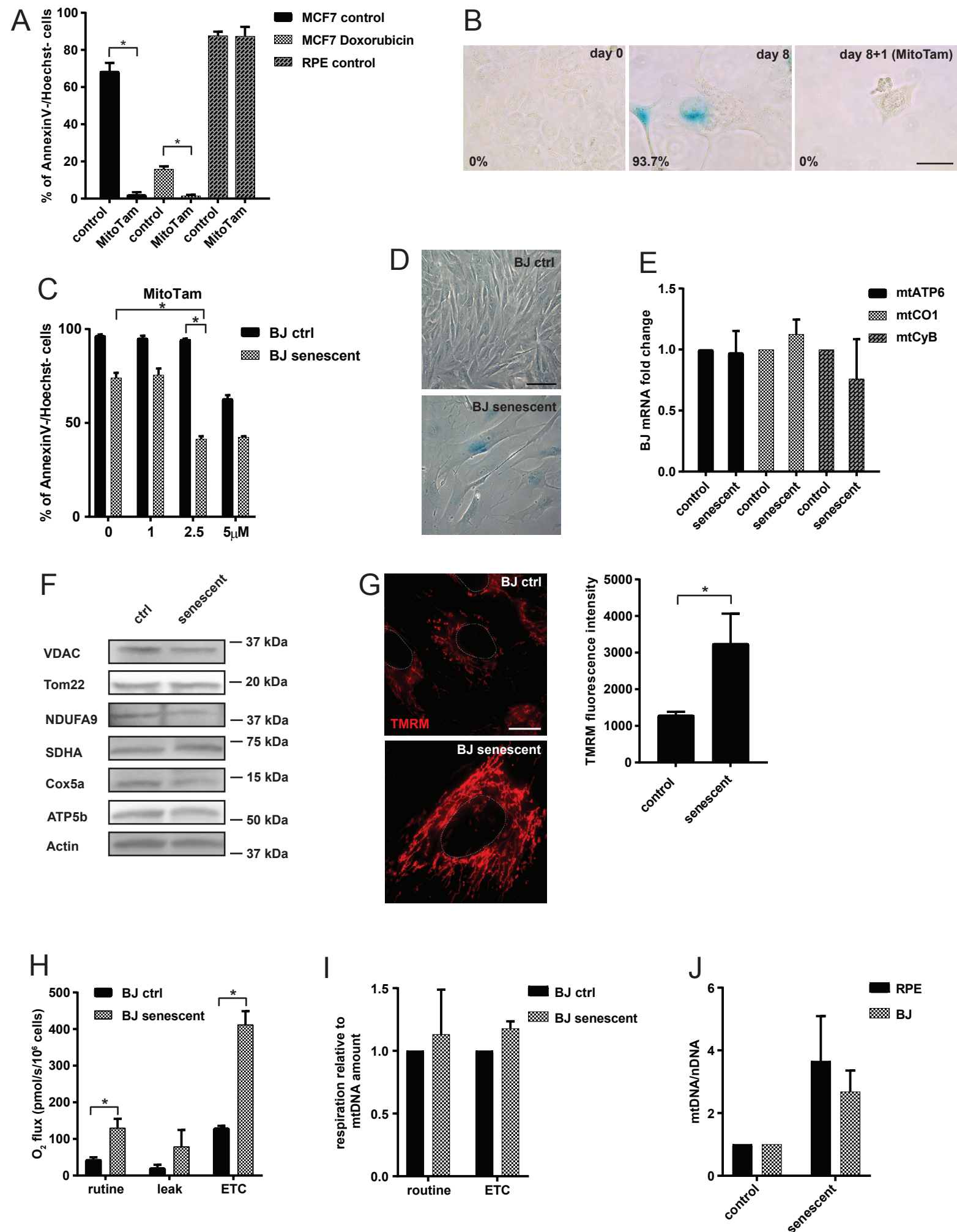

Supplement: Supplementary file 1 — Supplementary Figure 1 [file 41418_2018_118_MOESM1_ESM.pdf]

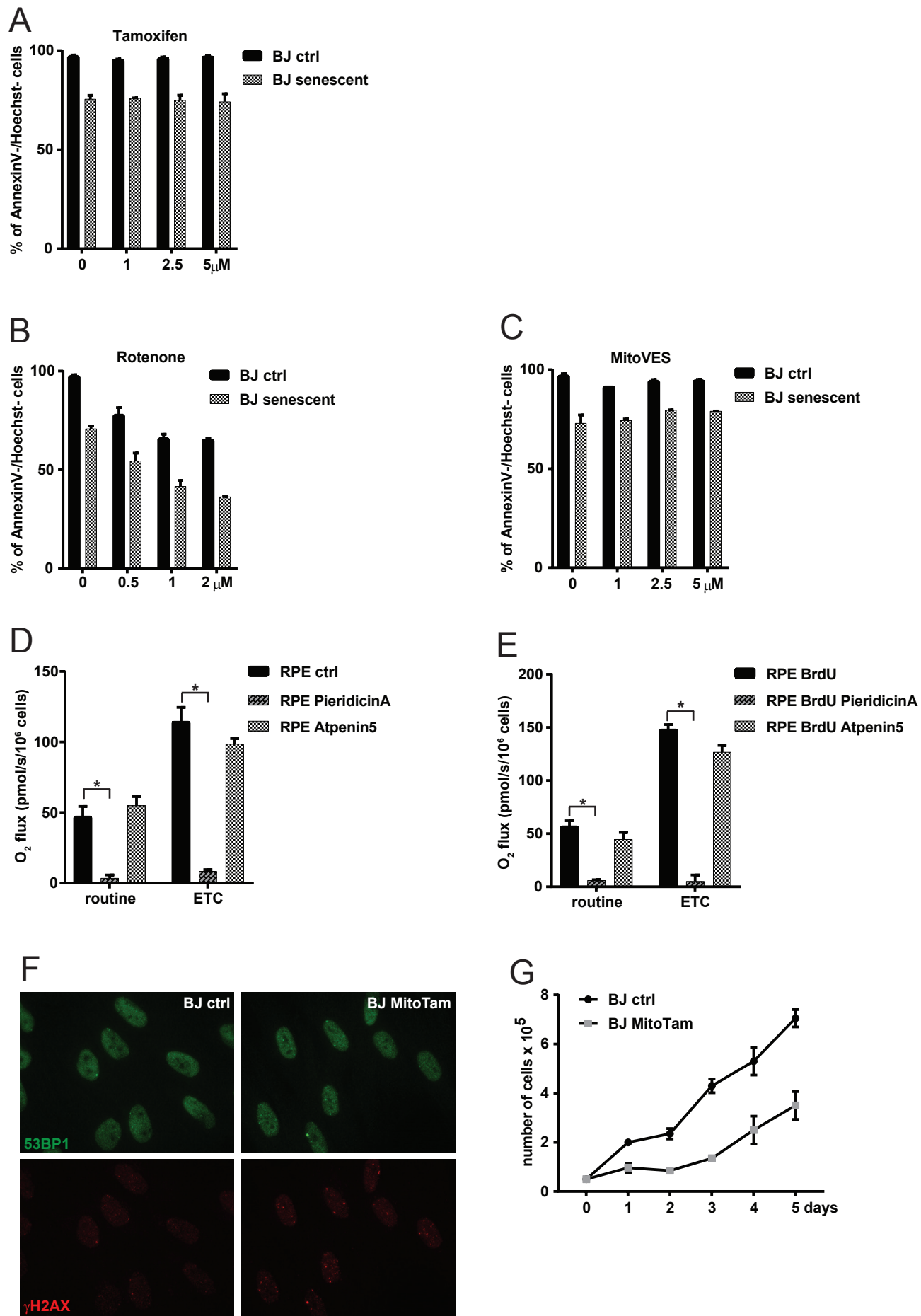

Supplement: Supplementary file 2 — Supplementary Figure 2 [file 41418_2018_118_MOESM2_ESM.pdf]

Suppl.Fig.3

A

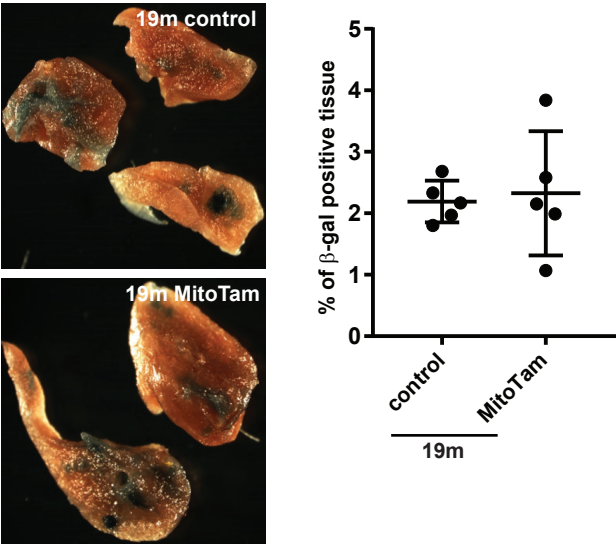

B

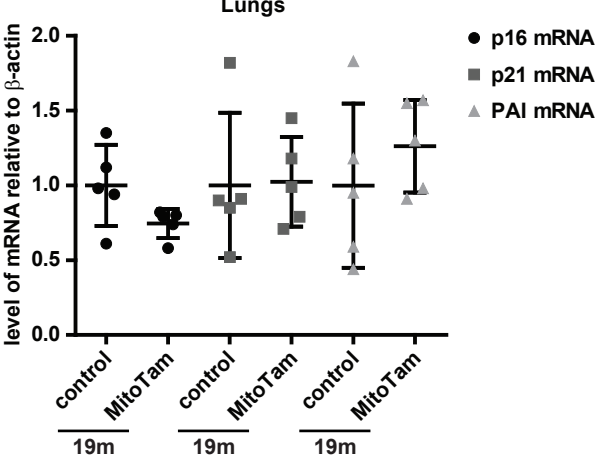

C

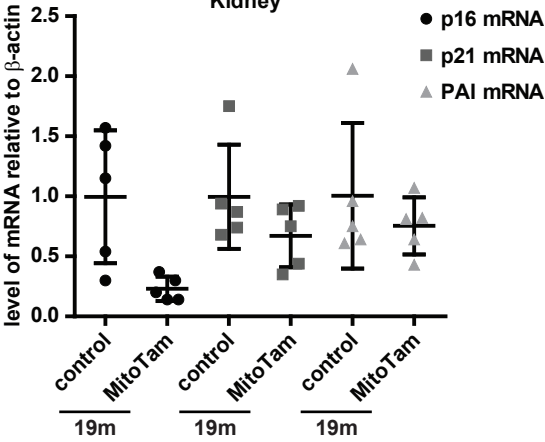

D

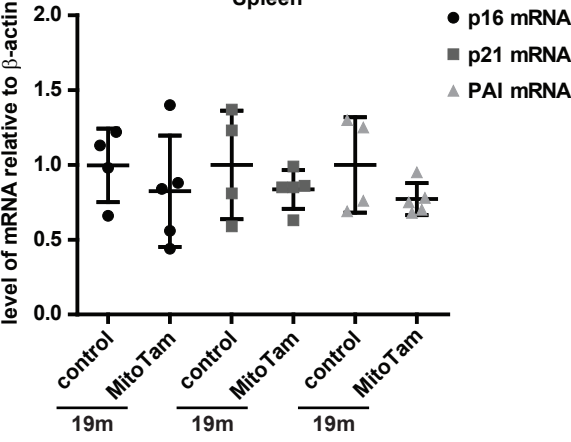

Supplement: Supplementary file 3 — Supplementary Figure 3 [file 41418_2018_118_MOESM3_ESM.pdf]

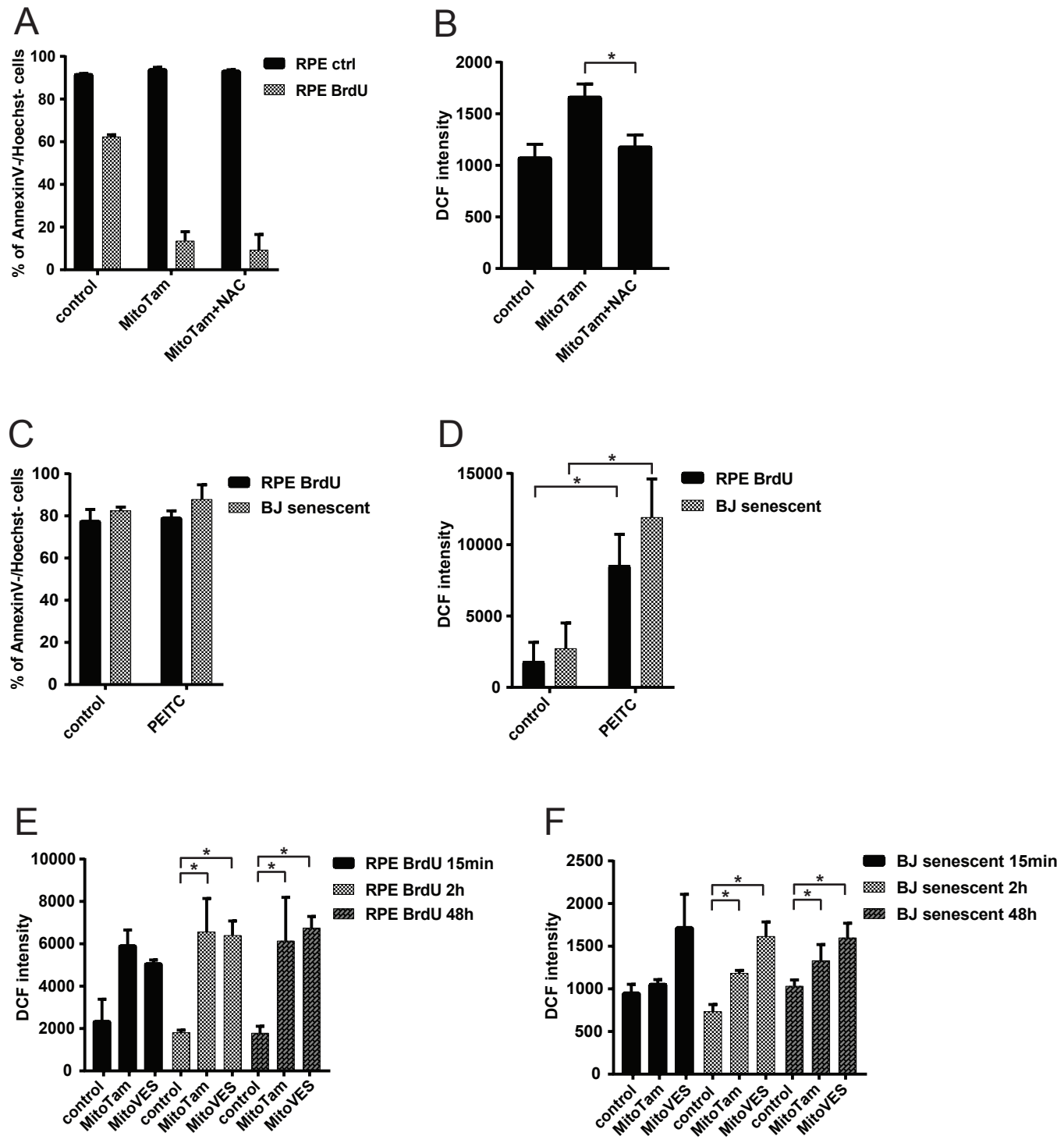

Supplement: Supplementary file 4 — Supplementary Figure 4 [file 41418_2018_118_MOESM4_ESM.pdf]

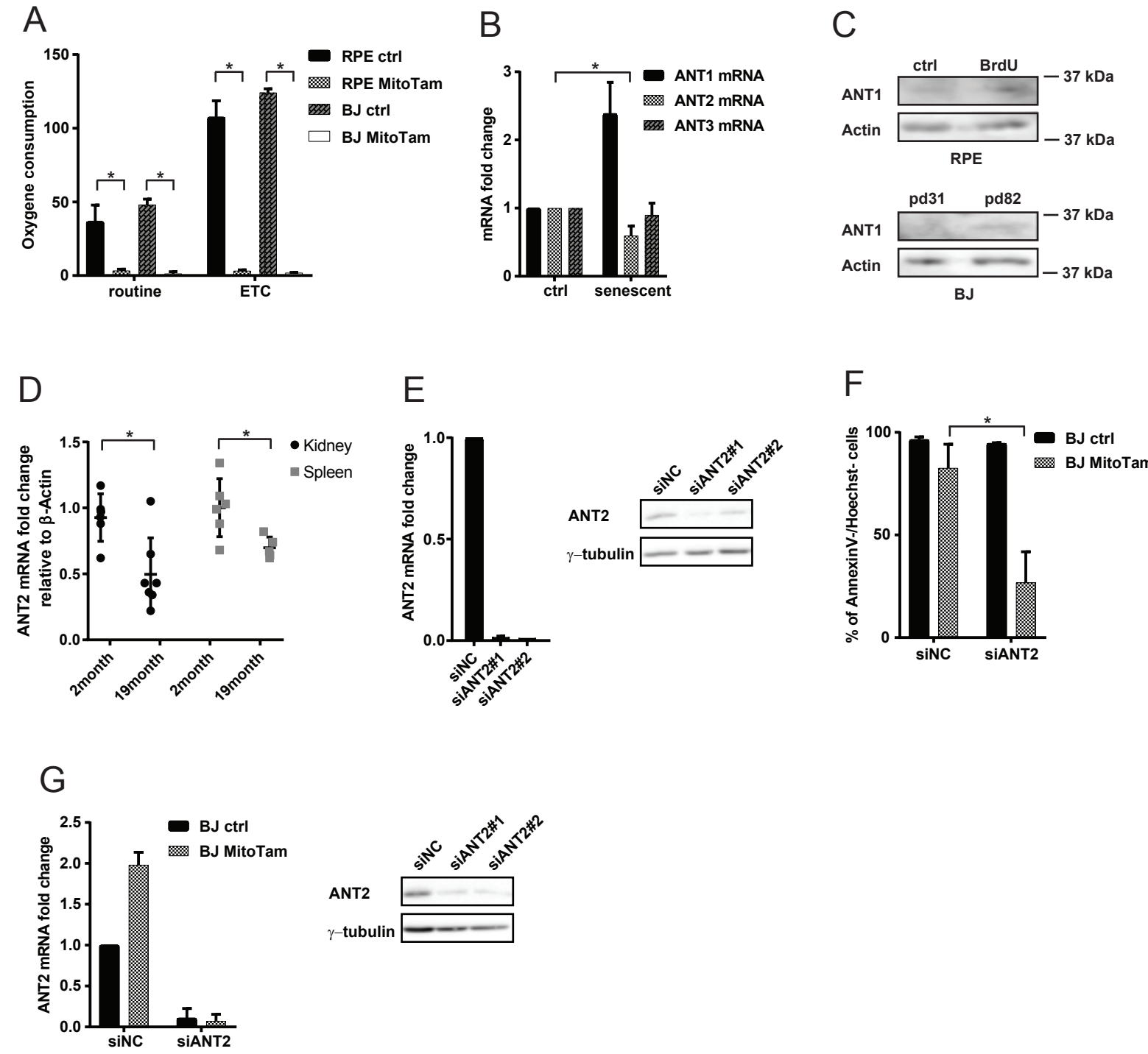

Supplement: Supplementary file 5 — Supplementary Figure 5 [file 41418_2018_118_MOESM5_ESM.pdf]

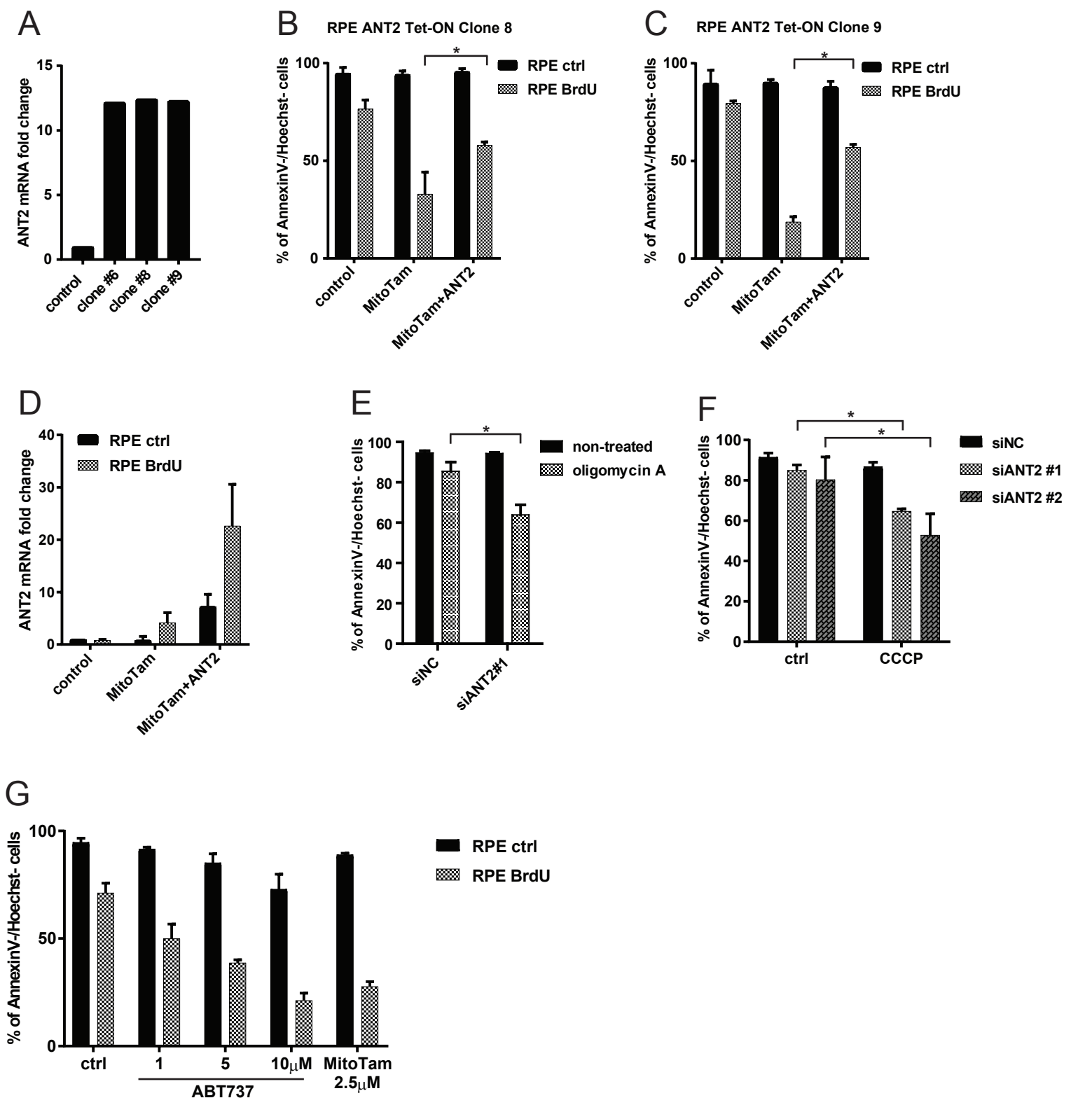

Supplement: Supplementary file 6 — Supplementary Figure 6 [file 41418_2018_118_MOESM6_ESM.pdf]
