# Supplementary material for: Selective elimination of senescent cells by mitochondrial targeting is regulated by ANT2
Source: Cell Death Differ. 2018 May 21;26(2):276–90. doi: 10.1038/s41418-018-0118-3 (PMC6329828; doi:10.1038/s41418-018-0118-3)
Supplement: Supplementary file 7 — Supplementary legend [file 41418_2018_118_MOESM7_ESM.docx]

**Supplementary Figure 1**

(A) Control and senescent MCF7 cells (treated with 0.75 μM doxorubicin for 8 days) were exposed to 1 μM MitoTam for 3 days, and cell survival was evaluated using the annexin V/Hoechst assay and flow cytometry. RPE cells were used as a control of toxicity. (B) MCF7 cells were treated with 0.75 μM doxorubicin for 8 days and then treated with 1 μM MitoTam for 24 h, and assessed for β-gal positivity. Scale bar represents 10 μm. (C) BJ control and senescent (replicative senescence) cells were exposed to MitoTam for 48 h at the concentrations as indicated and their survival was evaluated base on annexin V/Hoechst negativity using flow cytometry. (D) Control and replicative senescent BJ cells were assessed for β-gal positivity. Scale bar represents 50 μm. Control and replicative senescent BJ cells were assessed for expression of mtATP6, mtCO1 and mtCyB transcripts by qRT-PCR (E) and for VDAC, Tom22, NDUFA9, SDHA, Cox5a and ATP5b protein levels by immunoblotting with β-actin as a loading control (F). (G) Control and replicative senescent BJ cells were assessed for mitochondrial potential using TMRM and fluorescent microscopy (left panel) or flow cytometry (right panel). (H) Control and replicative senescent BJ cells were evaluated for routine, leak, and ETC respiration. (I) Respiration of RPE-1 control and senescent cells (BrdU) related to mtDNA amout. (J) Control and senescent RPE-1 and BJ cells were assessed for expression of mitochondrial (mtDNA) related to nuclear (nDNA) by qRT-PCR. Data in all graphs represent means ±S.D. from three independent experiments. Asterisk represents p< 0.05.

**Supplementary Figure 2**

BJ control and senescent (replicative senescence) cells were exposed to tamoxifen (A), rotenone (B) and MitoVES (C) for 48 h at the concentrations as indicated and their survival was evaluated by annexin V/Hoechst negativity using flow cytometry. RPE-1 control (D) and senescent cells (BrdU) (E) were treated with pieridicin A (20 μM) and Atpenin5 (20 μM) for 24 h and evaluated for routine and ETC respiration. (F) BJ control cells were treated with MitoTam (2.5 μM) for 48 h and DNA damage was assessed by immunofluorescence using 53BP1 and γH2AX proteins as markers. (G) BJ control cells were exposed to MitoTam (2.5 μM) for a period as indicated and assessed for proliferation. Data in all graphs represent means ±S.D. from three independent experiments. Asterisk represents p< 0.05.

**Supplementary Figure 3**

(A) 18 month old FVB/N mice were given one *i.p.* dose of MitoTam (2μg of MitoTam/1g of mouse) dissolved in 4% ethanol in corn oil or the excipient, lungs were excised and evaluated for β-gal positivity (blue colour). Level of p16, p21 and PAI transcripts in lungs (B) kidney (C) and spleen (D) were estimated by qRT-PCR. Asterisk represents p< 0.05.

**Supplementary Figure 4**

(A) Control and senescent (BrdU) RPE-1 cells were exposed to MitoTam (2.5 μM) for 48 h in the presence of N-acetylcystein (NAC; 1 mM), and cell survival was evaluated by annexin V/Hoechst negativity using flow cytometry; ROS levels in these cells were detected by flow cytometry using 2´,7´- dichlorofluorescein (DCF; 5 μM) (B). RPE-1 senescent (BrdU) cells and BJ senescent (replicative senescence) cells were exposed to PEITC (10 μM), and cell survival was evaluated by annexin V/Hoechst negativity using flow cytometry (C); levels of ROS in these cells were detected by flow cytometry as indicated above (D). RPE-1senescent (BrdU) cells (E) and BJ senescent (replicative senescence) cells (F) were exposed to MitoTam (2.5 μM) or MitoVES (2.5 μM) for 15 min, 2 h or 48 h, and the cells were evaluated for ROS levels as indicated above. Data in all graphs represent means ±S.D. from three independent experiments. Asterisk represents p< 0.05.

**Supplementary Figure 5**

(A) RPE-1 and BJ control cells were treated with MitoTam (2.5 μM) for 48 h and evaluated for routine and ETC respiration. (B) Expression of ANT1, ANT2 and ANT3 transcripts in BJ control and senescent (replicative senescence) cells was assessed by qRT-PCR. (C) Control and senescent (BrdU) RPE-1 cells, as well as BJ control (pd 31) and senescent (pd 82) cells were assessed for the level of the ANT1 protein by western blotting. β-actin was used as loading control. (D) Level of ANT2 transcript in kidney and spleen from 2 and 19 month old mice was estimated by qRT-PCR. (E) ANT2 transcript and protein level were evaluated by qRT-PCR and immunoblot in RPE-1 control cells 48 h after downregulation of ANT2 using specific siRNAs. (F) Control BJ cells were exposed to MitoTam (2.5 μM) for 24 h after downregulation of ANT2 and their survival was evaluated by annexin V/Hoechst negativity by flow cytometry. (G) ANT2 transcript and protein level were evaluated by qRT-PCR and immunoblot in BJ control cells 48 h after downregulation of ANT2 using specific siRNAs.

**Supplementary Figure 6**

(A) Control and ANT2-overexpressing RPE-1 cells (clones 6, 8 and 9) were assessed for the level of ANT2 mRNA by qRT-PCR. Control and ANT2-overexpressing RPE-1 cells (clone 8 (B) and clone 9 (C)) were exposed to MitoTam (2.5 μM) for 48 hand their survival was evaluated by annexin V/Hoechst negativity using flow cytometry. (D) Control and senescent (BrdU) RPE-1 cells as well as their ANT2-overexpressing counterparts were exposed to MitoTam (2.5 μM) for 48 h and assessed for the level of ANT2 mRNA using qRT-PCR. (E) Control RPE-1 cells were exposed to oligomycin A (5 μM) for 48 h after downregulation of ANT2 and their survival was evaluated by annexin V/Hoechst negativity by flow cytometry. (F) Control RPE-1 cells were exposed to CCCP (10 μM) for 48 h after downregulation of ANT2 and their survival was evaluated by annexin V/Hoechst negativity by flow cytometry. (G) Control and senescent (BrdU) RPE-1 cells were exposed to ABT737 for 48 h at concentration indicated, and their survival was evaluated by annexin V/Hoechst negativity by flow cytometry. 2.5 μM MitoTam was used as a control. Data in all graphs represent means ±S.D. from three independent experiments. Asterisk represents p< 0.05.
